# Detecting and Controlling DNA Translocation through a Nanopore using a van der Waals Heterojunction Diode

**DOI:** 10.21203/rs.3.rs-5193820/v2

**Published:** 2024-11-11

**Authors:** Sihan Chen, Siyuan Huang, Jangyup Son, Edmund Han, Kenji Watanabe, Takashi Taniguchi, Pinshane Y. Huang, William P. King, Arend M. van der Zande, Rashid Bashir

**Affiliations:** † Holonyak Micro and Nanotechnology Laboratory, University of Illinois Urbana-Champaign, Urbana, IL 61801, United States; ‡ Department of Mechanical Science and Engineering, University of Illinois Urbana-Champaign, Urbana, IL 61801, United States; ‡‡ Present addresses: Functional Composite Materials Research Center, Korea Institute of Science and Technology, Wanju-gun, Jeonbuk 55324, Republic of Korea; Division of Nanoscience & Technology, KIST School, University of Science and Technology (UST), Seoul 02792, Republic of Korea; Department of JBNU-KIST Industry-Academia Convergence Research, Jeonbuk National University, Jeonju, Jeonbuk 54896, Republic of Korea; # Department of Materials Science and Engineering, University of Illinois Urbana-Champaign, Urbana, IL 61801, United States; || Research Center for Electronic and Optical Materials, National Institute for Materials Science, Tsukuba, Ibaraki 305-0044, Japan; ¶ Research Center for Materials Nanoarchitectonics, National Institute for Materials Science, Tsukuba, Ibaraki 305-0044, Japan; § Materials Research Laboratory, University of Illinois Urbana-Champaign, Urbana, IL 61801, United States; †† Department of Biomedical and Translational Science, University of Illinois Urbana-Champaign, Urbana, IL 61801, United States; ¶¶ Department of Bioengineering, University of Illinois Urbana-Champaign, Urbana, IL 61801, United States; ## Chan Zuckerberg Biohub Chicago, Chicago, IL 60642, USA

**Keywords:** nanopore, van der Waals heterojunction, DNA, single molecule, ion transport

## Abstract

A long-unrealized goal in solid-state nanopore sensing is to achieve out-of-plane electrical sensing and control of DNA during translocation, which is a prerequisite for base-by-base ratcheting that enables DNA sequencing in biological nanopores. Two-dimensional (2D) heterostructures, with their capability to construct out-of-plane electronics with atomic layer precision, are ideal yet unexplored candidates for use as electrical sensing membranes. Here we demonstrate a nanopore architecture using a vertical 2D heterojunction diode consisting of p-type WSe_2_ on n-type MoS_2_. This diode exhibits rectified interlayer tunneling currents modulated by ionic potential, while the heterojunction potential reciprocally rectifies ionic transport through the nanopore. We achieve concurrent detection of DNA translocation using both ionic and diode currents and demonstrate a 2.3-fold electrostatic slowing of translocation speed. Encapsulation layers enable robust operation while preserving the spatial resolution of atomically sharp 2D heterointerface for sensing. These results establish a paradigm for out-of-plane electrical sensing and control of single biomolecules.

## Introduction

Nanopore sensors, with a sensing volume comparable to the analyte size, are a powerful tool for single-biomolecule analysis (1, 2). Seventeen years ago, IBM introduced the idea of out-of-plane electrical sensing and control using a DNA transistor (3). This design employs electrodes separated by a thin dielectric to control and sense DNA translocation through a nanopore. However, limitations in thin-film processes hindered the scaling of the transistor to the molecular thicknesses needed for sensing (4). Vertically stacked 2D heterostructures provide layer-by-layer control to construct out-of-plane electronics (5, 6), opening possibilities beyond single 2D materials for sensing with out-of-plane electric fields and interlayer currents. Unlike three-dimensional (3D) diodes with a depletion region, vertical 2D junctions exhibit an atomically sharp energy band discontinuity at their hetero-interface (6), which represents the ultimate limit for DNA transistors, with the conducting layers separated by only an angstrom-sized van der Waals gap. These attributes make vertical 2D heterojunction diodes an ideal yet unexplored candidate for use as electrical sensing membranes.

In this work, we present a nanopore architecture integrated with a vertical 2D heterojunction diode from p-type WSe_2_ on n-type MoS_2_, referred to as the HJD-NP sensor. We first electrically characterized the heterojunction to demonstrate the p-n diode behavior, then characterized the transport characteristics of both the ionic and diode channels in the absence of analytes, and finally demonstrated DNA sensing using an HJD-NP sensor. Three key advances from these proof-of-principle experiments include: (i) concurrent detection of DNA translocation through a vertical diode nanopore using both ionic and diode currents, (ii) slowing of DNA translocation via electrostatic interactions between the DNA and diode, and (iii) rectified ionic transport controlled by heterojunction potential. These results highlight the potential of HJD-NP sensors for sensitive single-biomolecule analysis with high spatiotemporal resolution.

## Results

### Nanopore integrated with a vertical 2D diode.

**Fig. 1.**
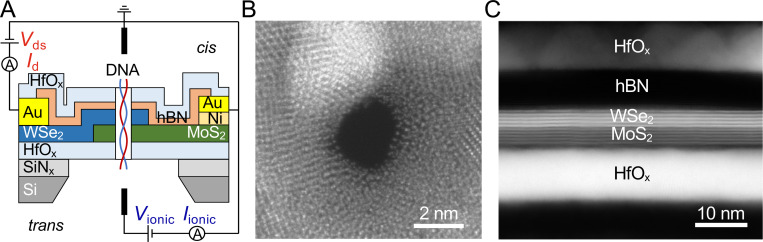
Device architecture and structural characterization. (*A*) Schematic of device architecture and measurement setup of an HJD-NP, with n-type MoS_2_ and p-type WSe_2_. When a double-stranded DNA molecule translocates through the nanopore, it induces changes in the interlayer current. (*B*) High-angle annular dark-field (HAADF) STEM image of an example nanopore drilled through a membrane stack. (*C*) Cross-sectional HAADF-STEM image of a representative device showing the membrane stack.

Fig. 1 presents the basic concept of the HJD-NP sensors for DNA sensing. As illustrated in Fig. 1A, a nanopore is formed in an electrically contacted 2D heterojunction, which consists of a p-type WSe_2_ flake stacked on an n-type MoS_2_ flake. This heterojunction forms an out-of-plane p-n diode. When a double-stranded DNA (dsDNA) molecule translocates through the nanopore under an external ionic bias *V*_ionic_, the sensor simultaneously interrogates the conventional ionic current *I*_ionic_ through the pore and the electrical current *I*_d_ across the diode under a drain-source bias *V*_ds_.

The detailed device fabrication procedures are described in the Methods and Supplementary Fig. S1. Briefly, we started the fabrication with HfO_x_ membranes formed by atomic layer deposition (ALD) (Supplementary Fig. S2), which is hydrophilic (7) and durable in salt solutions (8). Then we transferred 2–5 nm (3–7 layers) thick MoS_2_ and WSe_2_ flakes onto the membrane to form the heterojunction stack (6), with a 1–10 μm^2^ overlap region. Next, we fabricated n-type and p-type ohmic contacts to MoS_2_ and WSe_2_ using evaporated Ni/Au contacts and transferred Au contacts, respectively (Supplementary Fig. S3). Subsequently, we transferred a multiple hBN flake to fully encapsulate the WSe_2_/MoS_2_ heterostructure (9). This hBN buffer layer preserves the electrical conductivity of p-type WSe_2_ during ALD (10). Afterwards, we deposited a layer of ALD HfO_x_ for electrical insulation and stable operation. Finally, we drilled a nanopore in the overlap region through the membrane stack using a focused electron beam in scanning transmission electron microscopy (STEM) mode, which minimizes electron-beam induced damage to electronic materials compared to TEM mode (11). Fig. 1B shows the STEM image of an example nanopore. Further details on nanopore drilling are provided in the Supplementary Text 1 and Supplementary Fig. S4.

We confirmed the structure of the heterojunction and cleanliness of the interface between layers with cross-section STEM. Fig. 1C shows a representative membrane stack, which consists of HfO_x_, hBN, five-layer WSe_2_, five-layer MoS_2_, and HfO_x_ from top to bottom. The hetero-interface between WSe_2_ and MoS_2_ is atomically clean, which is critical for efficient interlayer charge carrier transport (12). Raman microscopy in Supplementary Fig. S5 further suggests interlayer coupling between WSe_2_ and MoS_2_.

**Fig. 2.**
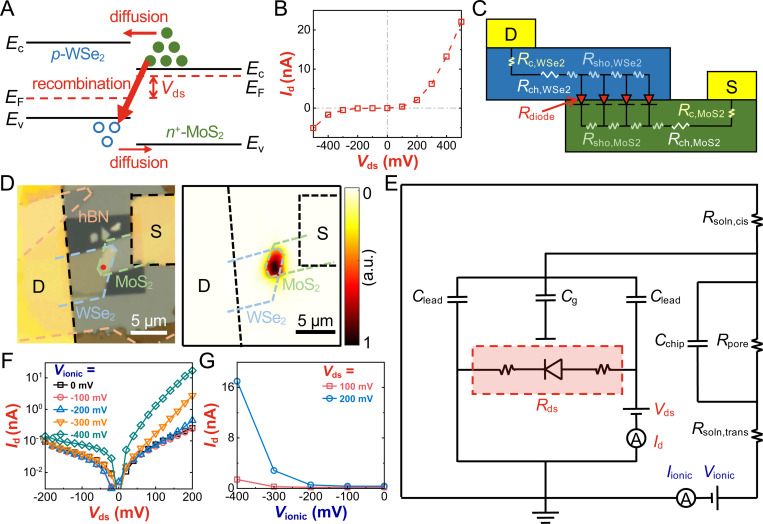
Equivalent circuit diagrams and electrical characterization. (*A*) The ideal band diagram of a vertical *p*-WSe_2_/*n*^+^-MoS_2_ diode under forward *V*_ds_ bias. The current due to interlayer recombination between the majority carriers of WSe_2_ and MoS_2_ dominates over the diffusion current (5, 15). (*B*) *I*_d_−*V*_ds_ curve of an example HJD-NP sensor measured in dry air in dark. (*C*) Equivalent circuit of the van der Waals heterojunction. *R*_c,WSe2_ and *R*_c,MoS2_ are the contact resistances to WSe_2_ and MoS_2_, respectively. *R*_ch,WSe2_ and *R*_ch,MoS2_ are the channel resistances of WSe_2_ and MoS_2_ in the non-overlapping region, respectively. *R*_sho,WSe2_ and *R*_sho,MoS2_ are the sheet resistances of WSe_2_ and MoS_2_ in the overlapping region, respectively. *R*_diode_ is the interface resistance between WSe_2_ and MoS_2_ per unit area. (*D*) Optical image (left) and corresponding photocurrent map (right) at *V*_ds_ = 0 mV of an example HJD-NP. The red dot indicates the location of the nanopore. (*E*) Equivalent circuit of an HJD-NP. *R*_soln,cis_ and *R*_soln,trans_ are the resistances of the salt solution in the *cis* and *trans* chambers, respectively, *C*chip is the chip capacitance, *R*_pore_ is the nanopore resistance, *C*_lead_ is the capacitance between the electrolyte and the contact leads on the drain or source side, , *C*_g_ is the capacitance between the electrolyte and the heterojunction, and *R*_ds_ is the total resistance of the heterojunction. (*F*) Output and (*G*) Transfer curves of the heterojunction in an HJD-NP under liquid gating in a 1 M LiCl buffer.

To use the heterojunction current for sensing, we must first understand the electrical transport properties and sensitivity of the van der Waals heterojunction. Due to defects incorporated during synthesis and environmental interactions (13, 14), few-layer WSe_2_ is lightly p-doped while few-layer MoS_2_ is heavily n-doped, as confirmed by FET transport measurements in Supplementary Fig. S3. Vertical stacking of *p*-WSe_2_ on *n*^+^-MoS_2_ forms a p−n diode with a type-II energy band alignment (Fig. 2A). Under forward bias, *I*_d_ is dominated by interlayer recombination between the majority carriers of WSe_2_ and MoS_2_ over diffusion current, and *I*_d_ is maximized when the hole density of WSe_2_ and the electron density of MoS_2_ are balanced (5, 15). Fig. 2B shows the *I*_d_−*V*_ds_ curve of an example HJD-NP sensor measured in dry air in dark, exhibiting forward rectification characteristic of a p-n diode.

However, unlike an ideal vertical p-n diode, the total van der Waals heterojunction resistance *R*_ds_ also includes series resistances (Fig. 2C), which consist of the contact resistances to WSe_2_ and MoS_2_, as well as the channel resistances of WSe_2_ and MoS_2_ in both the non-overlapping and overlapping regions. We establish that the p-n junction is the dominant resistance in the system using photocurrent microscopy (Supplementary Fig. S6A). Since photocurrent is sensitive to the electric gradient within the junction, its magnitude is greatest at the junction with the highest resistance. Fig. 2D shows the photocurrent map for an example device at *V*_ds_ = 0 mV. The strongest photo-response came from the MoS_2_/WSe_2_ overlap region, confirming the formation of a p-n junction (5). Supplementary Fig. S6B shows additional photocurrent maps at *V*_ds_ ≠ 0 mV for this device, which also supports this finding. Other heterojunction devices exhibited a strong photoresponse from the Au-WSe_2_ junction or the WSe_2_ channel region (Supplementary Fig. S6C,D) and/or non-rectifying *I*_d_−*V*_ds_ characteristics (Supplementary Fig. S7). We screened all diodes using photocurrent microscopy and selected those with both strong rectification and a dominant photocurrent in the overlap region for sensing measurements. Details on device yield are discussed in the Methods.

Next, we studied the device behavior in an ionic environment to identify the optimal measurement parameters for electrical sensing. There are two control knobs for device operation, namely *V*_ionic_ and *V*_ds_. Fig. 2E illustrates the coupling between the ionic and diode channels, which is comparable to that of FET-nanopore sensors (16–20), except here the sensing element is a vertical diode rather than a planar FET. The ionic voltage controls the heterojunction current transport via electrostatic gating. As shown in Fig. 1A, the backside HfO_x_ serves as the gate dielectric, while the front side is grounded to minimize electrochemistry. Fig. 2F,G shows the output and transfer characteristics of the heterojunction in an HJD-NP device, respectively. The current rectification improved with a more negative *V*_ionic_, indicating that the *p*-WSe_2_ partly limits the charge transport in the heterojunction and that the contact and channel resistances of the *n*^+^-MoS_2_ are comparably small. The FET transport characteristics of a backgated *p*-WSe_2_/*n*^+^-MoS_2_ heterojunction on a SiO_2_/Si substrate also supports these findings (Supplementary Fig. S8). Therefore, *p*-WSe_2_/*n*^+^-MoS_2_ heterojunction under forward bias can be modeled as a vertical p-n diode in series with an additional p-FET. We assume a uniform electric potential of *αV*_d_ for WSe_2_ and 0 mV for MoS_2_ in the overlap region, where *α* is the ratio of the vertical p-n diode resistance to the total heterojunction resistance. Consequently, applying a more negative *V*_ionic_ could further reduce the series resistances of the heterojunction in an HJD-NP, thereby enhancing the sensitivity of electrical sensing.

### Electrical rectification of ionic current.

**Fig. 3.**
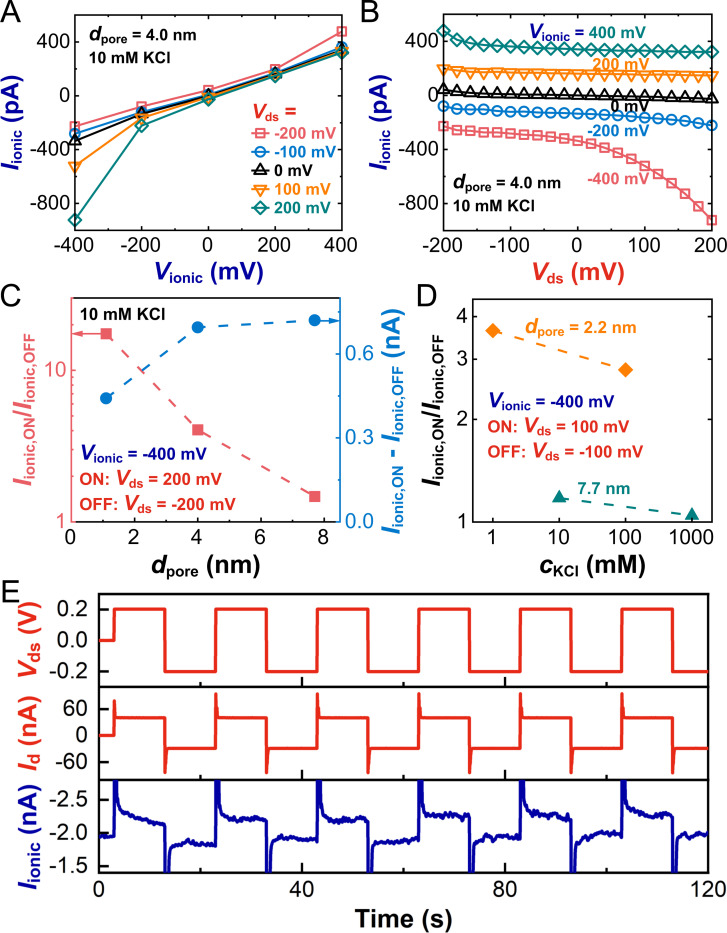
Ionic current modulation. (*A*) Output and (*B*) Transfer curves of the ionic transport of an HJD-NP with a *d*_pore_ of 4.0±0.3 nm under varying *V*_ds_. The heterojunction stack consists of 3.4 nm thick WSe_2_ on top of 3.8 nm thick MoS_2_. (*C*) Ionic current on/off ratio *I*_ionic,ON_/*I*_*i*onic,OFF_ and the maximum difference in ionic current *I*_ionic,ON_ - *I*_*i*onic,OFF_ versus *d*_pore_ at *V*_ionic_ = −400 mV in a 10 mM KCl buffer. ON state: *V*_ds_ = 200 mV; OFF state: *V*_ds_ = −200 mV. (*D*) *I*_ionic,ON_/*I*_*i*onic,OFF_ versus *c*_KCl_ at *V*_ionic_ = −400 mV for two nanopore devices. ON state: *V*_ds_ = 100 mV; OFF state: *V*_ds_ = −100 mV. (*E*) Response of *I*_d_ and *I*_ionic_ of an HJD-NP to cyclic switching of *V*_ds_ between ±200 mV at a fixed *V*_ionic_ of −400 mV. The pore size was 7.7±0.3 nm, and the buffer was 10 mM KCl.

Fig. 3 examines the opposite situation of how the diode potential affects ionic transport. Fig. 3A,B show the output and transfer curves of ionic transport of a liquid-gated HJD-NP with a pore size *d*_pore_ of 4.0±0.3 nm in 10 mM KCl. The ionic I–V characteristics were modulated by *V*_ds_. Specifically, (i) |*I*_ionic_| increased (decreased) when *V*_ds_ and *V*_ionic_ had opposite (the same) polarities. (ii) *I*_ionic_ exhibited greater variation with *V*_ds_ under forward bias compared to reverse bias. (iii) |*I*_ionic_| was most rectified at the most negative *V*_ionic_.

To understand the mechanism behind the ionic current control by *V*_ds_, we measured four HJD-NP sensors with *d*_pore_ from 1.1±0.3 nm to 7.7±0.3 nm and KCl concentrations *c*_KCl_ from 1 mM to 1 M (Supplementary Fig. S9). Fig. 3C plots the ionic current on/off ratio *I*_ionic,ON_/*I*_*i*onic,OFF_ and the maximum change in ionic current *I*_ionic,ON_ - *I*_*i*onic,OFF_ versus *d*_pore_ in a 10 mM KCl buffer, and Fig. 3D plots *I*_ionic,ON_/*I*_*i*onic,OFF_ versus *c*_KCl_ for two nanopore devices. The data shows that *I*_ionic,ON_/*I*_*i*onic,OFF_ decreased significantly with *d*_pore_ to the power of −1.25±0.10 and slightly with *c*_KCl_ to the power of −0.03 to −0.06. In addition, *I*_ionic,ON_ - *I*_*i*onic,OFF_ increased sub-linearly with *d*_pore_. Fig. 3E shows the cyclic changes in *I*_ionic_ as *V*_ds_ switched between ±200 mV at a fixed *V*_ionic_ of −400 mV, demonstrating stability and repeatability.

There are two potential mechanisms for modulating the ionic currents with interlayer potentials: (i) edge electrochemistry at the nanopore, and (ii) electrostatic interactions. If edge chemistry dominated, then *I*_ionic,ON_ - *I*_*i*onic,OFF_ would increase linearly with *d*_pore_ (21), which contradicts our observations. Additionally, *I*_ionic,ON_ - *I*_*i*onic,OFF_ for one HJD-NP sensor remained relatively constant as *c*KCl increased from 1 mM to 100 mM (Supplementary Fig. S10), confirming negligible electrochemistry.

After ruling out edge electrochemistry, we attribute the control of ionic transport by *V*_ds_ to electrostatic interactions between the biased heterojunction and the ions within the nanopore channel. There are two possible origins of electrostatic modulation of the ionic transport: (i) interlayer voltage drop across the vertical heterojunction (22, 23), and (ii) field-effect gating like an embedded electrode (24, 25). We ruled out the effect of the vertical potential drop, as reversing the heterojunction stacking sequence, and hence the direction of the electric field from *V*_ds_, did not reverse the induced ionic transport (Supplementary Fig. S9C,D). Therefore, we attribute the control of ionic transport by *V*_ds_ to field-effect gating, which effectively explains the observed modulation of ionic transport by *V*_ds_ (Supplementary Text 2) and can be leveraged for dynamic control of DNA translocation speed (26–29).

Additionally, we modelled ion transport in HJD-NP with a floating diode (Supplementary Text 3, Supplementary Table S1, and Supplementary Fig. S11). This ionic model was used to estimate *d*_pore_ in liquid measurements based on the measured ionic conductance. We also examined the effect of grounding the diode on ionic transport (Supplementary Fig. S12). The ionic I–V curves before and after grounding the diode remained linear and nearly identical, indicating negligible leakage and that connecting the diode with *V*_ds_ = 0 mV does not affect ion transport.

### Electrical sensing and control of DNA.

**Fig. 4.**
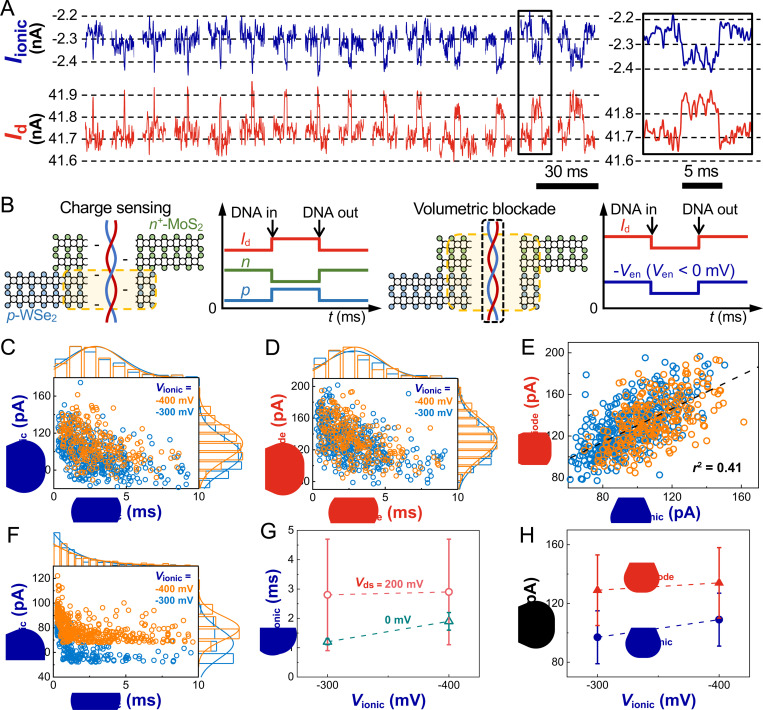
Concurrent diode and ionic current sensing of DNA translocation. (*A*) Concatenated signal traces (left) and zoom-in view of a single concurrent event (right). *V*_ionic_ = −300 mV, *V*_ds_ = 200 mV. (*B*) Two potential electrical sensing mechanisms for detecting translocating dsDNA through a *p*-WSe_2_/*n*^+^-MoS_2_ heterojunction nanopore: charge sensing and volumetric blockade-based sensing. In charge sensing, as negatively charged dsDNA enters the nanopore, it improves the balance between hole density *p* and electron density *n* at the hetero-interface, leading to an increase in *I*_d_. In volumetric blockade-based sensing, when a negative *V*_ionic_ is applied, the amplitude of the voltage at the nanopore entrance *V*_en_ decreases upon DNA translocation, resulting in a decrease in *I*_d_. The sensitive region is shown in golden zone. Scatterplots of the concurrent events detected in the (*C*) ionic channel and (*D*) diode channel, showing changes in ionic current ∆*I*_ionic_ and diode current ∆*I*_diode_, as well as dwell times for both ionic *τ*_ionic_ and diode *τ*_diode_ signals. *V*_ionic_ = −300, −400 mV, *V*_ds_ = 200 mV. (*E*) Signal amplitude correlation between concurrent events in the ionic and diode channels. *V*_ionic_ = −300, −400 mV, *V*_ds_ = 200 mV. (*F*) Scatterplots of the events detected in the ionic channel. *V*_ionic_ = −300, −400 mV, *V*_ds_ = 0 mV. (*G*) *τ*_ionic_ (median ± s.d.) versus *V*_ionic_. *V*_ds_ = 0, 200 mV. (*H*) ∆*I*_ionic_ and ∆*I*_diode_ (median ± s.d.) versus *V*_ionic_. *V*_ds_ = 200 mV. The heterojunction stack consists of 3.5 nm thick WSe_2_ on top of 2.0 nm thick MoS_2_. The pore size was 15±0.3 nm. The DNA molecule was 2,686 bp pUC19 plasmid. The electrolyte was 10 mM KCl in both cis and trans chambers.

In Fig. 4, we demonstrate the electrical sensing and control of dsDNA using an HJD-NP, with the measurement setup depicted in Fig. 1A. Both the *cis* and *trans* chambers were filled with 10 mM KCl. 50 nM of 2,686 bp circular dsDNA was added into the *trans* chamber. *V*_ionic_ was varied from −300 mV to −400 mV, while *V*_ds_ was varied from 0 mV to 200 mV. As expected, DNA translocation was detected only in the ionic channel at *V*_ds_ = 0 mV (Supplementary Fig. S13A,B). In contrast, DNA translocation was simultaneously detected in both ionic and diode channels at *V*_ds_ = 200 mV (Supplementary Fig. S13C,D). Fig. 4A shows example traces of these concurrently detected events. Both the ionic and diode currents increased upon DNA translocation. The increase of the ionic current is a signature of DNA translocation in dilute salt solutions like 10 mM KCl, where the transport of the counterions of DNA dominates over bulk ion transport (30, 31). The simultaneous increase of the diode current suggests the presence of DNA inside the nanopore channel reduced the diode resistance.

When a dsDNA molecule translocates through an HJD-NP, two non-capacitive electrical sensing mechanisms contribute to changes in *I*_d_ (Fig. 4B): (i) charge sensing and (ii) volumetric blockade-based sensing. The negative charges from the translocating DNA strand reduce the charge imbalance at the hetero-interface of *p*-WSe_2_/*n*^+^-MoS_2_, leading to an increase in *I*_d_ (Supplementary Text 4). Volumetric blockade-induced local potential change would otherwise lead to a decrease in *I*_d_, and therefore can be ruled out. The transconductance of this heterojunction was only 0.8 pA/mV (Supplementary Fig. S14). Based on analytical calculations and simulations (16, 18), we estimate a maximum potential change of 1 mV at the nanopore entrance, corresponding to a change in *I*_d_ of < 1 pA. No capacitive signals were observed in the ionic or diode channel. Since *R*_lead_ exceeded 10 GΩ (Supplementary Fig. S15) and *C*_lead_ was only 9.2 pF (Methods), both capacitive crosstalk and direct leaking between the ionic and diode channels should be negligible (17, 18). Above all, we ascribe the observed increase in diode current upon DNA translocation to charge sensing.

A total of 409 concurrent events were detected at *V*_ionic_ = −300 mV, *V*_ds_ = 200 mV and 238 events at *V*_ionic_ = −400 mV, *V*_ds_ = 200 mV, as shown in the scatterplots in Fig. 4C,D. The current changes of the concurrent events detected in the ionic channel ∆*I*_ionic_ and the diode channel ∆*I*_diode_ are weakly correlated, with a Pearson’s correlation coefficient of *r*^2^ = 0.41 (Fig. 4E), suggesting different physical origins. Fig. 4F shows the scatterplots of the events detected in the ionic channel at *V*_ionic_ = −300, −400 mV, *V*_ds_ = 0 mV. The median and standard deviation (s.d.) of the dwell times and current changes were extracted from these scatterplots and presented in Fig. 4G,H. The details of data processing and analysis are in the Methods. Notably, the median dwell time at *V*_ionic_ = −300 mV increased from 1.2 ms to 2.8 ms as *V*_ds_ increased from 0 mV to 200 mV. As *V*_ionic_ changed from −300 mV to −400 mV, ∆*I*_ionic_ increased by 12%, from 97±18 pA to 109±18 pA, while ∆*I*_diode_ increased by only 4%, from 129±24 pA to 134±24 pA. The ionic signal-to-noise ratio (SNR) was 3.5–3.6, while the diode SNR was 3.9–4.2. This diode SNR is comparable to the FET SNR of 2D FET-nanopore sensors (Supplementary Table S2).

Interestingly, the dwell time increased with a more negative *V*_ionic_ and a more positive *V*_ds_. In addition, these dwell times in the range of 0.5–1.1 bp/μs are about two orders magnitude slower than dsDNA translocation through an Al_2_O_3_ nanopore of similar size (32). We ascribe slowed DNA translocation to the field-effect gating of the nanopore channel by the biased heterojunction, as the effective gate potentials with respect to the WSe_2_ and MoS_2_ at the nanopore channel, i.e., |*V*_ionic_ - α*V*_d_| and |*V*_ionic_|, both increased with a more negative *V*_ionic_ and a more positive *V*_ds_. The gating effect on DNA could be significant even when the pore size is much larger than the Debye length (27, 28). The slight increase in ∆*I*_ionic_ with |*V*_ionic_| is consistent with previous studies in dilute salt solutions (33, 34). The relatively small increase in ∆*I*_diode_ with |*V*_ionic_| further supports the charge sensing mechanism, as the effective charge of DNA does not change with *V*_ionic_ (35).

## Discussion

We demonstrated electrical sensing of DNA translocation through a nanopore using the intralayer current of a vertical 2D diode, concurrently observing slowed transport due to electrostatic interactions between the DNA and diode. This heterojunction nanopore platform enables sensing capabilities that exceed the limitations of ionic and electrical sensing with single 2D materials. Exciting future directions include exploring band-to-band tunneling with type-III band alignment and measuring correlated intralayer currents within each semiconductor layer to complement interlayer measurement. Additional strategies could further improve the diode’s SNR and slow DNA translocation, such as minimizing series resistances with degenerate doping (36, 37), reducing background noise by decreasing the overlap region between WSe_2_ and MoS_2_, and enhancing sensitivity and control with a smaller nanopore in a thinner membrane stack. Collectively, the vertical 2D diode nanopore presents promising pathways toward solid-state nanopore sequencing.

## Materials and Methods

### HJD-NP device fabrication.

Supplementary Fig. S1 illustrates the fabrication procedure. The process started with a 20±2 nm thick commercial SiNx membrane (NBPX4001Y-LR, Norcada) with a 10×10 μm window size on a 4 mm large, 10–20 Ω cm low-resistivity silicon substrate. A lightly doped silicon substrate was chosen over an intrinsic silicon substrate to prevent electrostatic discharge, which could further reduce the already low yield (38). Next, (i) a 10–15 nm thick HfO_x_ layer was deposited onto the front side of the SiN_x_ membrane at 200 °C using ALD (Savannah S100 ALD system). The thickness of the HfOx layer was measured using ellipsometry (J.A. Woollam VASE). Following this, (ii) the SiNx was etched from the HfO_x_/SiN_x_ membrane stack using a 60 s XeF_2_ gas etch (XACTIX XeF_2_ etching system) at 3 Torr (Supplementary Fig. S2), with the substrate placed upside down on a clean glass slide during etching. ALD HfO_x_ served as the etch stop. Consequently, the supporting membrane was converted from 20 nm SiN_x_ to 10–15 nm HfO_x_, which offers improved wettability (7) and durability (8).

Subsequently, (iii) 2–5 nm thick MoS_2_ and WSe_2_ flakes were exfoliated from synthetic crystals (HQgraphene), stacked together, and transferred onto the membrane using the pick-up technique (9). The thickness of exfoliated 2D flakes (MoS_2_, WSe_2_, and hBN) was optically identified after exfoliation and verified with atomic force microscopy (AFM) after transfer. The size of the WSe_2_/MoS_2_ overlap region was 1–10 μm^2^. AFM tip-based cleaning was used to remove surface residues from the transfer process (39). Next, (iv) a 100×100 μm large, 50 nm thick gold pad was transferred onto the WSe_2_ flake as the p-type contact electrode in a N_2_-filled glovebox (40). Subsequently, (v) 5 nm Ni/30 nm Au was deposited onto the MoS_2_ flake as the n-type contact electrode using optical or ebeam lithography followed by ebeam evaporation. Then, (vi) a 5–20 nm thick hBN flake was exfoliated and transferred onto the membrane to fully encapsulate the WSe_2_/MoS_2_ heterostructure using the pick-up technique (9). This multilayer hBN flake reduces electron doping and damage to WSe_2_ from ALD HfO_x_. Next, a gentle remote O_2_ plasma process (1 min, 10 W, Tergeo plasma cleaner) was used to nucleate the hBN surface for complete and uniform ALD coverage (41). Afterwards, (vii) a 10–15 nm thick HfO_x_ layer was deposited onto the front side of the device at 200 °C using ALD for electrical insulation. Leakage tests in Supplementary Fig. S15A,B found a minimum of 10 nm ALD HfO_x_ was sufficient for electrical insulation. The total thickness of the final membrane stack (HfO_x_/hBN/WSe_2_/MoS_2_/HfO_x_) was 30–60 nm.

Finally, (viii) a 1–15 nm nanopore was drilled through the membrane stack in the WSe_2_/MoS_2_ overlap region using a focused electron beam at 300 kV in STEM (Themis Z, Thermo Fisher Scientific). A beam current of 13 nA (4 nA) was required to drill through the membrane stack within a few minutes in nanoprobe (microprobe) mode with spot size 1, corresponding to a beam size of about 1 nm (a few nm). The electron irradiation dose for initial imaging and locating the drilling spot was 10^7^ e^-^/nm^2^ (10^6^ e^-^/nm^2^). Additional details on nanopore drilling are provided in the Supplementary Text 1 and Supplementary Fig. S4. After drilling, the nanopore was imaged in STEM with a 30 pA beam current and an atomic beam size (nanoprobe mode with spot size 9).

### Cross-sectional STEM sample fabrication and imaging.

A protective layer of 5–30 nm thick amorphous carbon was thermally evaporated onto the nanopore device membrane. The cross-sectional STEM sample was then fabricated using standard focused ion beam (FIB) lift-out procedures with a FIB-SEM system (Helios 600i DualBeam, Thermo Fisher Scientific). A cryo-can was used during FIB thinning to minimize redeposition. The cross-sectional sample was imaged using an aberration-corrected STEM (Themis Z, Thermo Fisher Scientific), operated at 300 kV with a convergence angle of 25.2 mrad. Elemental mapping was performed using the Super-X EDS detector.

### Photocurrent mapping.

Scanning photocurrent measurements were performed by rastering a focused laser spot across the device surface, using a source measure unit (2450 SourceMeter, Keithley) for voltage sourcing and a current preamplifier (SR570, Stanford Research System) for current measurement. The laser spot had a diameter of 1 μm, a wavelength of 488 nm, and a power of 70 μW.

### Raman measurements.

Raman measurements were performed with a confocal Raman microscope (Nanophoton Raman 11) using a 532 nm laser with a 100× objective. Raman spectra were obtained using a grating of 2400 l mm^−1^ at a laser power of 0.5 mW/cm^2^ and an acquisition time of 30 s. Raman maps were obtained using a grating of 2400 l mm^−1^ at a laser power of 0.5 mW/cm^2^, an acquisition time of 3 s/pixel and a pixel size of 300 nm.

### Nanopore measurements and data analysis.

Before the experiment, the fabricated chip was attached onto a custom-made printed circuit board (PCB) by applying silicone elastomer (Kwik-cast, World Precision Instruments) around the edges of the chip. The silicone elastomer was then further applied around the membrane to reduce the chip capacitance and provide additional insulation between the metal leads and the electrolyte, leaving an exposed area of less than 0.01 mm^2^. *C*_lead_ was 5.2 nF without the silicone paint and 9.2 pF with the silicone paint, measured using triangular *V*_ionic_ waves (42). Afterwards, the chip was electrically connected to the PCB by wire bonding. The PCB was then sandwiched between two chambers of mechanically clamped custom-made PMMA flow cells.

Next, both chambers were sequentially flushed with de-ionized (DI) water (18 MΩ cm, Milli-Q, Millipore), IPA , DI water, and a salt solution of 10 mM KCl, 10 mM Tris, 1 mM EDTA at a pH of 7.4±0.2. Both the DI water and salt solution were degassed overnight using a dry scroll vacuum pump (SVF-E3M-20PC, Scroll Labs), which is critical for successful nanopore wetting. In most cases, the nanopore could not be fully wetted immediately. To promote wetting, three methods were employed: (i) flushing both chambers with IPA, (ii) leaving both chambers with degassed 10 mM KCl solution overnight, and (iii) ramping the ionic voltage between ±500 mV in 10 mM KCl.

All current measurements were performed using an integrated patch-clamp amplifier (MultiClamp 700B, Axon Instruments), with channel 1 dedicated to the ionic channel and channel 2 to the diode channel. Channel 1 was connected to a pair of Ag/AgCl electrodes to apply *V*_ionic_ and measure *I*_ionic_. The ionic voltage was applied to the *trans* chamber, while the *cis* chamber was grounded. Channel 2 was connected to the PCB to apply *V*_ds_ and measure *I*_d_. The electrical voltage was applied to the drain side with p-WSe_2_, while the source side with n-MoS_2_ was grounded. Current offsets in both channels were adjusted to zero at zero biases. The entire setup was housed in a Faraday cage with a dedicated low-noise ground connection on a vibration isolation table.

After complete nanopore wetting, both *V*_ionic_ and *V*_ds_ were varied while measuring *I*_ionic_ and *I*_d_ to study the interactions between the ionic channel and the diode channel. Additional leakage tests in Supplementary Fig. S15C,D established the operation limits of HJD-NPs as |*V*_d_ - *V*_ionic_| ≤ 600 mV and |*V*_s_ - *V*_ionic_| ≤ 600 mV. The sampling rate was 1 kHz, and the data were low-pass-filtered at 20 Hz using the built-in 8-pole Bessel filter. The output signal was digitized by a Digidata 1440A (Axon Instruments) and recorded using pClamp 10.7 software. Finally, for DNA sensing experiments, 50 nM of 2,686 bp circular dsDNA (pUC19 plasmid, New England Biolabs) was added to the *trans* chamber. The sampling rate for DNA sensing was 100 kHz. A 2 kHz low-pass 8-pole Bessel filter was applied to both channels for event detection and statistical analysis. Blank experiments were conducted at the applied voltages before DNA insertion. Event detection was performed using the open-source Matlab code package Transalyzer (43). Events shorter than 0.25 ms were excluded, and concurrent events were identified when the event start time in both channels differed by 0.5 ms or less. *τ*_ionic_ and *τ*_diode_ were determined using the full-width, half-maximum (FWHM) values of the event (43). ∆*I*_ionic_ and ∆*I*_diode_ were determined by dividing the event charge deficit by the FWHM time (43). *τ*_ionic_ and *τ*_diode_ were analyzed statistically by fitting the event dwell time histogram with a Gaussian peak for *V*_ds_ = 200 mV and an exponential decay for *V*_ds_ = 0 mV. ∆*I*_ionic_ and ∆*I*_diode_ were analyzed statistically by fitting the event amplitude histogram with a Gaussian peak.

### Yield.

We began the fabrication process with a total of 111 SiN_x_ membranes. The first 30 devices were used to optimize the fabrication protocol. After optimization, 56 reached the final fabrication step. Out of the fabricated devices, 35 (63%) were excluded from liquid measurements due to an insensitive WSe_2_/MoS_2_ overlap region, as determined by both I-V characteristics and photocurrent mapping. 21 devices reached the liquid measurement stage, but 13 of these failed to fully wet. We managed to perform DNA translocation measurements on 8 devices and successfully detected concurrent events in both ionic and diode channels in 1 device. This low yield aligns with previous challenges in fabricating FET-nanopore devices (16, 44–47).

## Supplementary Material

Supplement 1
