# Supplementary material for: Detecting and Controlling DNA Translocation through a Nanopore using a van der Waals Heterojunction Diode
Source: Res Sq. 2024 Nov 11:rs.3.rs-5193820. Preprint. [Version 2] doi: 10.21203/rs.3.rs-5193820/v2 (PMC11601842; doi:10.21203/rs.3.rs-5193820/v2)
Supplement: Supplement 1 [file NIHPPRS5193820V2-supplement-1.pdf]

Supplementary Files

This is a list of supplementary files associated with this preprint. Click to download.

- HJDnpsiv9preprint.pdf
